# Antiapoptotic Role for Lifeguard in T Cell Mediated Immune Response

**DOI:** 10.1371/journal.pone.0142161

**Published:** 2015-11-13

**Authors:** Tatiana Hurtado de Mendoza, Fei Liu, Inder M. Verma

**Affiliations:** Laboratory of Genetics, The Salk Institute for Biological Studies, La Jolla, California, United States of America; Icahn School of Medicine at Mount Sinai, UNITED STATES

## Abstract

Anti-apoptotic protein Lifeguard (LFG) is upregulated on T cells upon *in vitro* activation. To investigate its role in T cell immunity we infected wild type and LFG knockout bone marrow chimaeras mice with LCMV. We observed a decreased number of LFG KO activated CD8 and CD4 T cells throughout the infection and a marked decrease in LFG KO LCMV specific memory T cells. WT and KO T cells proliferated at the same rate, however, LFG KO CD44^hi^ T cells showed increased cell death during the initial phase of the immune response. LFG KO and WT T cells were equally sensitive to the FAS antibody Jo-2 in *ex vivo* cultures, and blocking extrinsic pathways of cell death *in vivo* with Fas L or caspase 8 inhibitors did not rescue the increased apoptosis in LFG KO T cells. Our data suggest that LFG plays a role in T cell survival during the initial phase of anti-viral immune response by protecting pre-existing memory T cells and possibly newly activated T cells resulting in a diminished immune response and a decreased number of LCMV specific memory T cells.

## Introduction

Lifeguard (LFG, also known as Faim2) was first discovered in our laboratory during a screen for molecules that can inhibit FAS mediated cell death. Though LFG expression is ubiquitous, highest expression was found in the brain [1]. It has been shown at least in tumor cell lines, that LFG expression is regulated by the Akt/LEF-1 pathway, conferring tumor cells resistance to apoptosis [2].

During an immune response to viral infection, T cell apoptosis intricately balances the proliferation of T cells at every phase of the immune response to ensure effective control of the virus without causing damage to the host. During the priming phase of a virus infection, especially ones that induce strong pro-inflammatory cytokine storms such as Lymphocytic Choriomeningitis virus (LCMV), attrition of pre-existing memory T cells precedes the expansion of virus specific T cells [3,4]. After the expansion phase, during which T cells expand 10^^4^ to 10^^5^ fold [5], 90% to 95% of the effector T cells undergo apoptosis, and the intrinsic apoptotic pathway has been shown to be the main mechanism [6]. Even in the relatively stable memory phase, memory T cells are maintained by a balance of homeostatic proliferation and apoptosis [7]. Given the role of LFG in protection against apoptosis, we hypothesize that LFG may play a role in anti-viral T cell immunity. Here we report that LFG confers protection to pre-existing memory T cells during the attrition process and possibly to recently activated T cells, resulting in a diminished immune response and memory T cell pool.

## Materials and Methods

### Mice

LFG KO, RAG1 KO (B6.129S7-Rag1tm1Mom/J) and CD45.1 B6 (B6.SJL-Ptprca Pep3b/BoyJ) mice were purchased from The Jackson Laboratory. All animal work was approved by the Institutional Animal Care Committee of the Salk Institute.

### Bone marrow chimaeras and LCMV infection

Bone marrow was extracted from femurs of CD45.1 WT and CD45.2 LFG KO mice, mixed in a 1:1 ratio and transplanted (10x10^6^ cells per mouse) by tail vein injection into lethally irradiated (11 Gray) RAG1 KO hosts. Eight weeks after bone marrow transplant mice were infected by intraperitoneal injection of LCMV Armstrong (2x10^5^ plaque-forming units) to induce an acute infection.

### *Ex vivo* proliferation assay and peptide stimulation

Flat-bottom 96 well plate were coated with purified CD3 antibody (eBioscience, San Diego) (10 ng/mL) over night at 4°C. Splenocytes from WT and KO mice were labeled with 1μM CFSE for 7 minutes and plated to the 96 well plate at 1x10^6^ cells/well with purified CD28 (eBioscience, San Diego) (2μg/mL) and incubated 1 to 3 days at 37°C. Splenocytes from virus infected chimera mice were cultured in the presence of 10^-6^M GP33 and NP396 and 5 μg/ml Brefeldin A for 6 hours at 37°C before intra-cellular staining for Interferon-gamma.

### Flow cytometry antibodies

We collected the spleen of mice at various timepoints after infection, splenocytes were harvested and stained with the following fluorophore conjugated antibodies: anti-CD45.1 (A20), anti-CD45.2 (104), anti-CD4 (L3T4), anti-CD8a (53–6.7), anti-CD44 (IM7), anti-Bcl2 (eBioscience, San Diego). Results were collected using the FACSDiva software in a LSRII (Becton Dickinson). Data was analyzed with the FlowJo software.

### TCR stimulation and LFG staining

Splenocytes were isolated and plated in a 96 well plate at 10^6^ cells per well. For TCR stimulation, non-tissue culture treated wells were previously coated with anti-CD3 (145-2C11) antibody at 5μg/ml and then CD28 antibody (37.58) was added to the media at 1μg/ml (BD Biosciences). After 16 hours cells were surface stained with CD8 and CD4 antibodies (4 wells were combined for staining) and then treated with cytofix-cytoperm solution (BD Biosciences) for 15 minutes at 4°C in the dark. Cells were washed two times in Perm Wash solution (eBioscience) and incubated for 15 minutes with mouse Fc-Block (eBioscience) followed by either LFG antibody (Imgenex) or rabbit IgG isotype control (DA1E, Cell signaling) diluted in Perm Wash 5% goat serum at 2.5μg/well. The secondary antibody was goat-anti-rabbit conjugated to Alexa 488 (Molecular Probes).

### Proliferation and cell death studies

In order to study proliferation *in vivo* we intraperitoneally injected 1 mg of BrdU per mouse the day before sacrificing them and used the BrdU Flow kit (BD Biosciences) to stain the splenocytes in combination with anti-CD45.1, anti-CD45.2, anti-CD4 and anti-CD8. For cell death studies we used phycoerythrin-conjugated Annexin V (BD Biosciences) and DAPI together with anti-CD45.1, anti-CD45.2, anti-CD4 and anti-CD8.

### *In vivo* blocking of the Fas pathway

On the day of LCMV infection mice also received an intraperitoneal injection of either control IgG, 100μg/mouse, in 2% DMSO, anti-FasL antibody, 100μg/mouse, (MFL3) (BD Biosciences) or caspase 8 inhibitor at 1mg/kg (Z-IETD-FMK) (BD Pharmingen). The following day the mice received again the same doses of IgG, MFL3 or Z-IETD-FMK. At day 2 p.i. mice were euthanized and their splenocytes were harvested and stained for CD45-1, CD45-2, CD4, CD8, CD44 and AnnexinV in order to evaluate if the compounds used were able to reduce cell death compared to the IgG control condition.

## Results

### LFG expression is enriched in T cells and it is upregulated upon TCR stimulation

**Fig 1:**
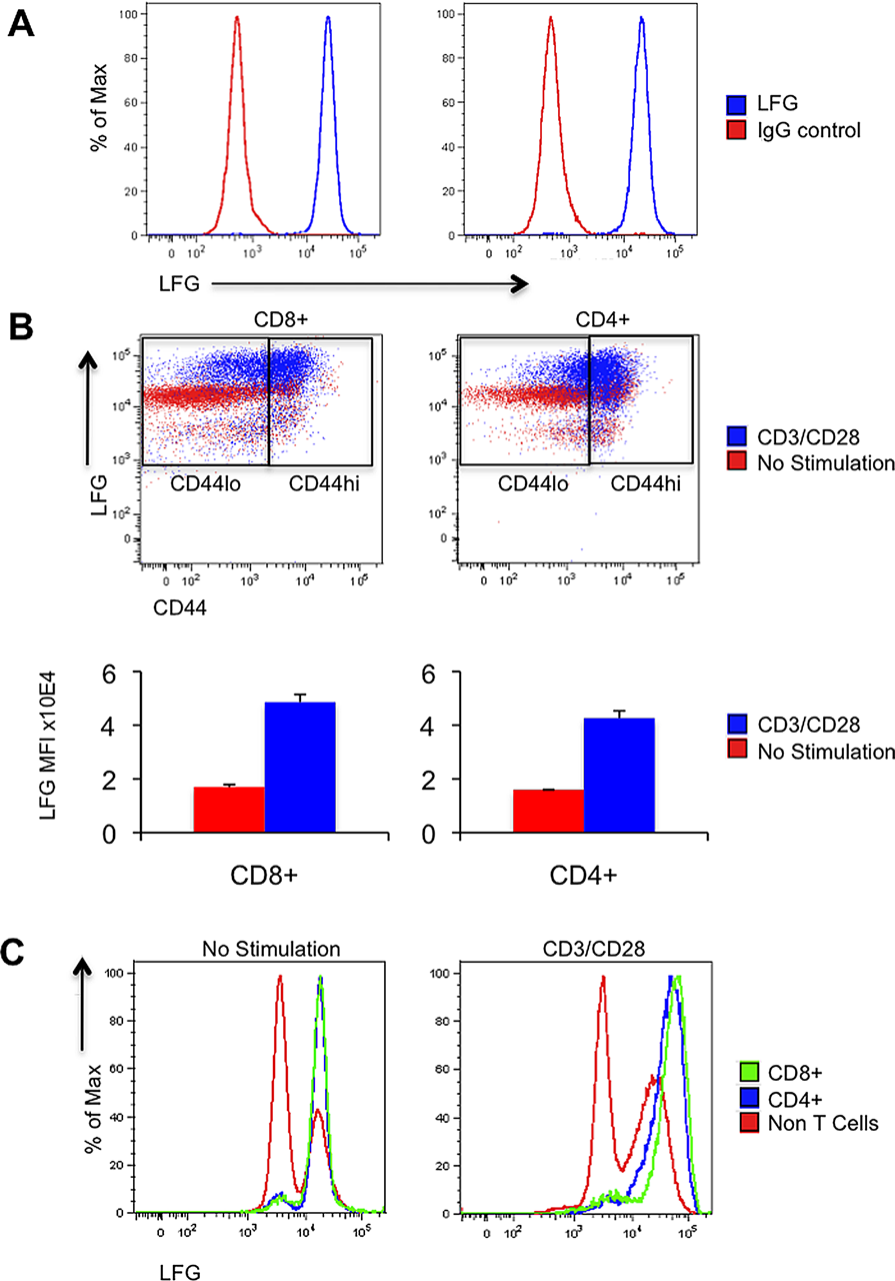
LFG is expressed in T cells and upregulated upon TCR stimulation. (A) Histograms showing LFG staining in blue compared to IgG control in red. The left panel is gated on CD8 T cells and the right panel on CD4 T cells. (B) FACS plots representing LFG and CD44 expression before (red) or after TCR stimulation by adding anti-CD3/CD28 (blue) in CD8 (left panel) and CD4 (right panel) T cells. The CD44^hi/lo^ gate was created according to the unstimulated condition. The bottom panel represents the values of LFG mean fluorescence intensity for CD8 or CD4 T cells in the no stimulation versus the CD3/CD28 condition. (C) Histograms representing LFG expression in CD4 (blue), CD8 (green) and non-T cells (red) before and after CD3/CD28 stimulation. (n = 4 mice, *p ≤ 0.05, **p ≤ 0.01, ± = SEM).

Since the discovery of LFG in our laboratory, we have previously focused on studying the tissue in which it was expressed at highest level, namely, the brain [8]. Given the importance of apoptosis in immunity, we also examined LFG expression on various types of cells of the immune system, and found that CD8 and CD4 T cells have the highest expression level of LFG (Fig 1A). In addition, upon activation by CD3/CD28, T cells can further increase the level of LFG expression. As shown in Fig 1B, before stimulation, only a small portion of the CD4 or CD8 T cells were CD44^hi^ (the CD44^hi/lo^ gate was drawn based on this pre-stimulation divide). After stimulation, as expected, almost all of the T cells have increased their CD44 expression, and the majority of the cells are now in the CD44^hi^ gate. We can clearly observe that stimulation induced upregulation of LFG expression in most of the T cells by almost a three-fold increase in mean fluorescence intensity. When we compared LFG expression between the T cells and non-T cells, it is clear that upregulation of LFG is specifically due to CD3 stimulation (Fig 1C). In the pre-stimulation condition T cells were already expressing higher levels of LFG than the rest of the splenocytes except for a small population represented by a second peak in the non-T cell population. Upon TCR engagement the most significant shift in LFG expression corresponded to the CD8 and CD4 T cell peaks with a 2.93 and a 2.68 fold increase from the unstimulated condition respectively, while the main peak of the non-T cells did not change (Fig 1C).

### LFG KO T cells showed a reduced T cell response and memory pool

**Fig 2:**
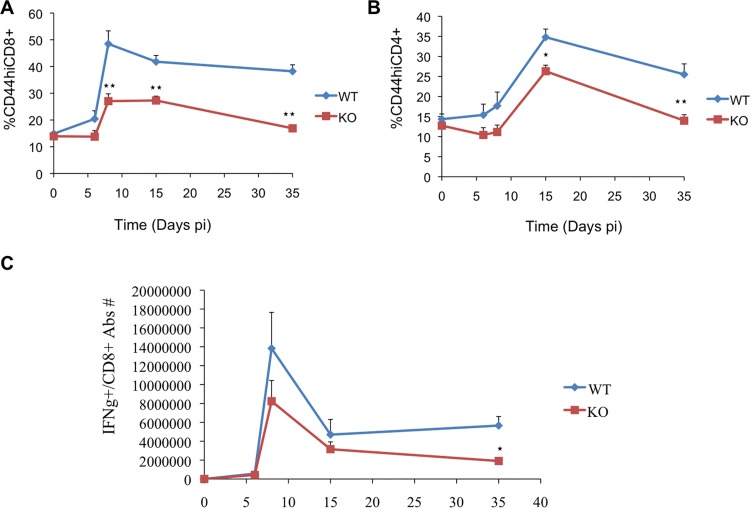
Decreased immune response of LFG KO T cells after LCMV infection. (A) The graphs represent the percentage of WT (diamonds) and KO (squares) CD44^hi^CD8 T cells (top panel) or CD44^hi^CD4 T cells (bottom panel) over the time course of the immune response to LCMV infection. We evaluated the pre-infection phase (day 0), expansion phase (days 6 and 8), contraction phase (day 15) and memory phase (day 35). (B) Decreased LFGKO antigen specific CD8 response to LCMV infection. The graph represent the combined absolute number of WT (diamonds) and KO (squares) CD8 T cells that are specific to NP396 and GP33 over the time course of the immune response to LCMV infection. Splenocytes from the infected chimera mice were stimulated with the two peptides for 6 hours in the presence of BFA and intracellularly stained for Interferon gamma. (n = 4 mice per timepoint, *p ≤ 0.05, **p ≤ 0.01, ± = SEM).

The upregulation of LFG expression upon CD3 stimulation prompted us to study the effect of the loss of LFG in antiviral immune response using a model of LCMV infection. For this purpose we generated bone marrow chimeras by mixing CD45.1 wild type and CD45.2 LFG KO bone marrow cells in a 1:1 ratio and transplanted them into lethally irradiated Recombination-Activating Gene 1 (Rag1) KO mice. Eight weeks after bone marrow transplant, mice were infected with LCMV Armstrong strain to induce an acute viral infection. We monitored the immune response of WT and KO CD8 and CD4 T cells during the expansion, contraction and memory phases. Without infection, WT and KO cells reconstituted the CD4 and CD8 population equally well at 8 weeks post-adoptive transfer (Fig 2, time point 0). However, upon infection, T cells lacking LFG became dominated by WT cells, especially CD8 T cells. Fig 2A shows that at day 8 p.i. the percentage of LFG KO CD44^hi^CD8 T cells was significantly reduced (48.46% +/- 4.87% for WT, as compared to 27.04% +/- 2.74% for KO (p = 0.0086)). These differences persisted at day 15 p.i. with 41.8% +/- 2.33% for WT and 27.3% +/- 1.61% for KO (p = 0.0022) and day 35 p.i. with 38.21% +/- 2.43 in WT and 16.88% +/- 0.53% in KO (p = 0.00014). LFG KO CD4 T cells were also at a disadvantage, but to a lesser extent. Among CD44^hi^/CD4 T cells we observed a decrease in the LFG KO population at day 15 p.i. with 34.79% +/- 2.02% for WT and 26.31% +/- 1.5% for KO (p = 0.015), and day 35 p.i. with 25.51% +/- 2.62% in WT and 13.97% +/- 1.48% in KO (p = 0.0087) (Fig 2B right panel). We also stimulated virus specific CD8 T cells directly *ex vivo* from the infected chimera mice with a mixture of the two most dominant LCMV peptides GP33 and NP396 (Fig 2C). By intracellular cytokine staining for IFN-g, we evaluated the number of peptide specific CD8 T cells over the course of infection, and found that WT CD8 T cells showed a more robust immune response than the KO ones. But more importantly we observed a three fold decrease in LCMV specific CD8 memory T cells (p = 0.01) (Fig 2C). Taken together, these data show that LFG KO cells have a reduced immune response to LCMV infection that is most significantly affecting, but not limited to, the memory pool of virus specific cells.

### LFG KO T cells have normal proliferation but are more sensitive to apoptosis

The decreased immune response observed in LFG KO cells can be attributed to defects in proliferation and/or survival. Therefore we measured apoptosis and proliferation in the initiation (Day 3 p.i.), expansion (Day 6,8 p.i.), contraction (Day 15 p.i.) and memory phases (Day 35 p.i.). In order to study proliferation we pulsed the mice with BrdU for a day and measured the percentage of proliferating CD8 and CD4 T cells in the spleen at each time point. Apoptosis was measured by Annexin V staining coupled with CD4 and CD8 staining.

**Fig 3:**
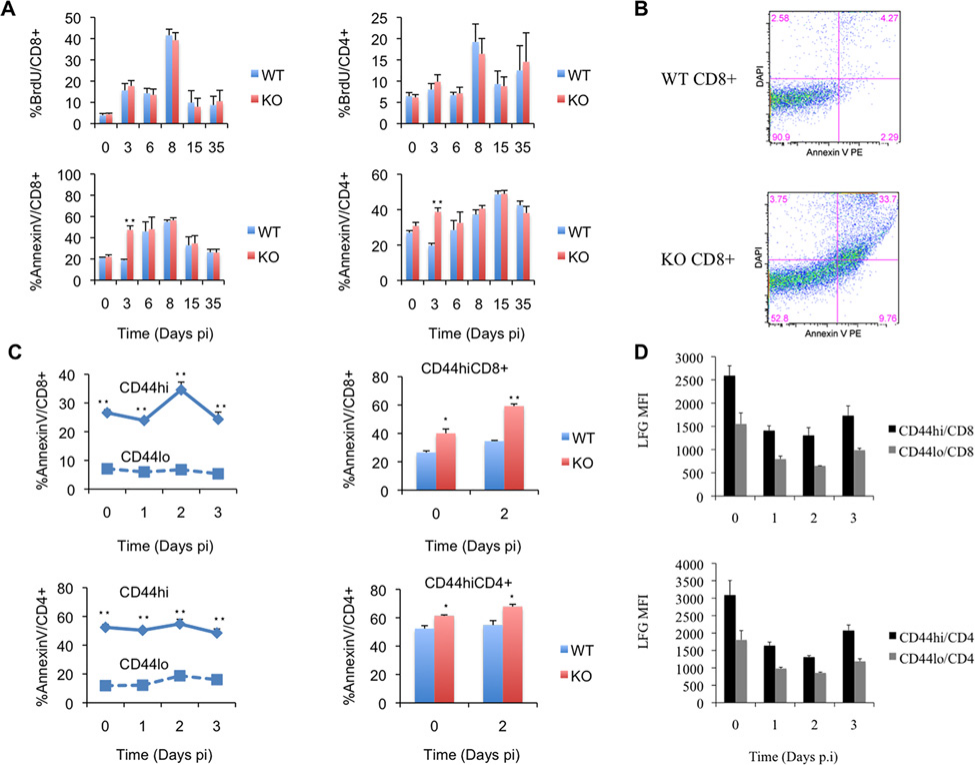
Apoptosis and proliferation studies. (A) In the top panel the bar graphs represent proliferation of CD8 (left) or CD4 (right) WT and KO T cells by measuring BrdU incorporation at the pre-infection (day 0), initiation (day 3), expansion (days 6 and 8), contraction (day 15) and memory (day 35) phases of the immune response. In the bottom panel the bar graphs represent apoptotic WT and KO T cells measured by AnnexinV staining at the same timepoints described above. (B) Dot plots representing AnnexinV and DAPI staining in WT or KO CD8+ T cells at day 3 p.i. (C) The graphs in the left represent the percentage of AnnexinV^+^ CD8 (top) or CD4 (Bottom) T cells in WT mice divided in CD44^hi^ (solid line, diamonds) and CD44^lo^ (dashed line, squares) over a timecourse from day 0 to day 3 p.i. in order to monitor cell death in the initial activation phase. The right panel shows bar graphs representing the percentage of apoptotic WT and KO cells by AnnexinV staining of CD44^hi^ CD8 (top) and CD4 (bottom) T cells at days 0 and 2 p.i. (D) Bar graphs representing the mean fluorescence intensity of LFG staining in CD44hi and lo CD8 and CD4 T cells at days 0 to 3 p.i. (n = 4 mice per timepoint, *p ≤ 0.05, **p ≤ 0.01, ± = SEM).

We did not find any significant differences in BrdU positive CD8 or CD4 T cells in any of the time points analyzed (Fig 3A Top panel). This finding is supported by our *ex vivo* CD3 stimulated proliferation assay (S1 Fig). We labeled WT and KO splenocytes with CFSE and activated them with plate-bound CD3 and soluble CD28 for 1 to 3 days. The proliferation profile demonstrated by serial dilution of CFSE intensity was superimposable between the WT and KO cells. These finding led to our conclusion that LFG KO cells had no proliferative disadvantage. On the other hand, we found a marked increase in Annexin V positive CD8 and CD4 KO T cells at day 3 p.i. (Fig 3A Bottom panel). In the CD8 population 18.75% +/- 1% of the WT cells were apoptotic, compared to 47.42% +/- 3.79% of the KO cells (p = 0.00033) (Fig 3B). Within the CD4 T cells we saw 19.72% +/- 1.25% apoptotic cells in the WT population and 38.72% +/- 2.26% in the KO (p = 0.00032). This observation actually fits well with our initial observation that the dominance of wild type over the KO cells occurred as early as day 6 post infection, especially for CD8 cells (Fig 2A). These data suggest that LFG’s role in T cell anti-viral immune response is not through proliferation, but rather through protection from apoptosis. This protection was not afforded during the contraction phase of the immune response, where the mass majority of the apoptotic events during the immune response occur, but rather during the initial priming phase of the immune response, which none-the-less had defining quality for the rest of the immune response phases.

### LFG protects CD44^hi^ T cells from death in the initial phase of the immune response

Apoptosis during the priming phase of LCMV infection has been previously reported, and has been termed memory attrition [3,4,9]. When we performed a more detailed time course study from day 0 to day 3 p.i. and looked at apoptosis in correlation with the CD44 status of the CD8 and CD4 T cells in wild type mice, we observed what was completely in agreement with previous reports. AnnexinV staining was predominant in the CD44^hi^ population, peaking at day 2 p.i. with 34.57% +/- 2.7% in CD44^hi^CD8 versus 6.77% +/- 0.5% in CD44^lo^CD8 cells (p = 5.5x10^-5^) and 54.8% +/- 3.16% in CD44^hi^CD4 compared to 18.72% +/- 0.98% in CD44^lo^CD4 cells (p = 3.57x10^-5^) (Fig 3C). We concluded that we needed to analyze apoptosis in combination with CD44 staining in our WT/KO bone marrow chimaeras at day 2 p.i. to find out whether the apoptosis in KO cells was also more severe in CD44^hi^ cells. We compared naïve mice with day 2 p.i. and found that the infection further increased the difference in apoptosis between wild type and knockout in the CD44^hi^ population. Wild type CD44^hi^CD8 cells at day 0 had 26.6% +/- 1.09% of apoptotic cells compared to 40.1% +/- 3% in the KO (p = 0.013). At day 2 p.i. we found 34.57% +/- 2.7% AnnexinV^+^ cells in the WT versus 59.32% +/- 2.12% in the KO CD44^hi^CD8 population (p = 0.00036). For CD44^hi^CD4 cells at day 0, 52.4% +/- 2.01% of the WT cells were apoptotic versus 61.3% +/- 0.7% of the KO cells (p = 0.014) and at day 2 p.i. we found 54.8 +/- 3.16% and 67.7 +/- 1.68% in WT and KO CD44^hi^CD4 cells respectively (p = 0.011) (Fig 3C). On the other hand we found no significant differences in cell death between the WT and KO CD44^lo^ population in either CD8 or CD4 T cells. These data shows that apoptosis is preferentially increased in LFG KO CD44^hi^ T cells during the initial phase of the immune response to LCMV infection and this effect is additive to the already high memory attrition phenomenon previously reported.

In addition we evaluated the expression of LFG from naïve to day 3 post-infection and found that LFG’s expression was highest in naïve mice, then decreased at days 1 and 2 and started to increase at day 3 p.i. These data could correlate with LFG expression decreasing at days 1 and 2 for the peak of cell death and increasing at day 3 maybe expressed as a survival signal in the recently activated T cells. Another important point is that LFG’s expression was higher in CD44hi T cells, which are the cells that undergo apoptosis in the LFG KO context (Fig 3D).

### LFG protects T cells from apoptosis in a Fas independent manner

**Fig 4:**
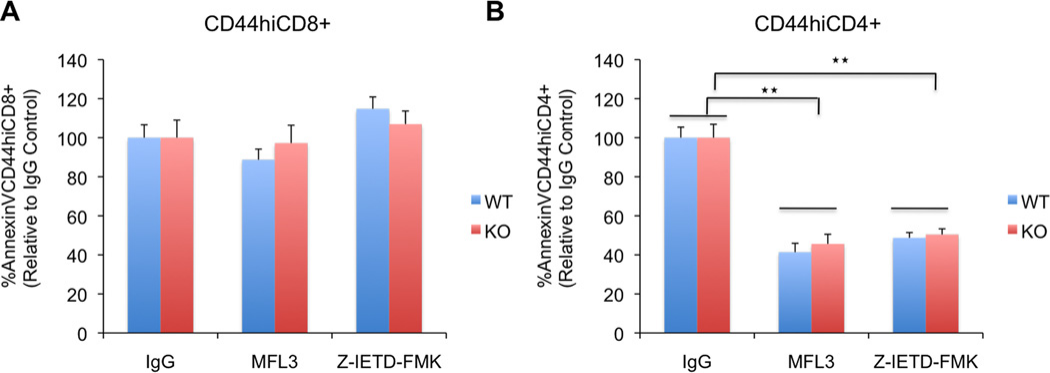
LFG protects T cells from apoptosis in a Fas independent manner. (A) (B) Bar graphs representing the percentage of apoptotic WT and KO CD44^hi^CD8 (A) or CD44^hi^CD4 T cells (B) in LCMV infected bone marrow chimaeras at day 2 p.i. that have been treated with IgG control, antibody to FasL (MFL3), or caspase 8 inhibitor (Z-IETD-FMK). The results are normalized to the IgG control that represents 100% of apoptosis. (n = 5 mice per condition, *p ≤ 0.05, **p ≤ 0.01, ± = SEM).

Since LFG was first discovered as a molecule that inhibits Fas induced apoptosis [1] we decided to use antibodies to FasL and caspase 8 inhibitors to block this pathway and see if we could rescue LFG KO cells from cell death. For this purpose the bone marrow chimaeric mice were injected intraperitoneally with IgG control antibody, anti-FasL antibody (MFL3) or the irreversible caspase 8 inhibitor Z-IETD-FMK on day 0 and day 1 p.i.. Mice were euthanized at day 2 p.i. in order to analyze cell death in the CD44^hi^CD8 and CD44^hi^CD4 populations. The results are expressed in percent AnnexinV^+^ cells relative to the IgG control mice that were normalized to 100%. We did not observe a significant reduction of cell death in CD44^hi^CD8 cells in either genotype. We found 88.7% +/- 5.47% (WT) and 97.26% +/- 9.12% (KO) cell death compared to IgG control for MFL3 (NS) and 114.7% +/- 6.1% (WT) and 106.99% +/- 6.66% (KO) for Z-IETD-FMK (NS) (Fig 4A). On the other hand CD44^hi^CD4 cells did show a significant reduction in apoptosis compared to the IgG control. Mice injected with MFL3 had 41.35% +/- 4.56% (WT) and 45.63% +/- 4.92% (KO) AnnexinV^+^CD44^hi^CD4 cells compared to IgG control (p = 0.0053 (WT), p = 0.0019 (KO)) and mice injected with Z-IETD-FMK had 48.75% +/- 2.7% (WT) and 50.41% +/- 2.93% (KO) (p = 4.09x10^-5^ (WT), p = 0.00017 (KO)) (Fig 4B). These data suggested that the Fas pathway was involved in the death of CD44^hi^CD4 cells but played no role in the death of CD44^hi^CD8 cells. However, the reduction in cell death observed in the CD44^hi^CD4 cells of the mice treated with FasL antibody or caspase 8 inhibitor was very similar for WT and KO, indicating that this pathway was not the one responsible for the increased apoptosis observed in the LFG KO cells.

In order to confirm that LFG was acting in a Fas independent manner in this context, we decided to test the sensitivity of WT and KO T cells to the Fas agonist Jo2, so we cultured splenocytes from naïve (day 0) or day 2 p.i. chimaeric mice in the presence or absence of Jo2. The results in Table 1 show that at day 0 both WT and KO CD44^hi^CD8 cells were resistant to Jo2. Upon infection, IgG treated KO cells showed significantly higher rates of apoptosis compared to WT, indicating that infection itself provides an environment in which KO CD44^hi^CD8 T cells are at a survival disadvantage. Addition of Jo2 increased cell death similarly (10%) in WT and KO cells leading us to conclude that there is no increased sensitivity of LFG KO CD44^hi^CD8 T cells to Fas mediated cell death. CD44^hi^CD4 T cells were responsive to Jo2 at day 0 and day 2 p.i., but apoptosis was increased by similar rates in both WT and KO cells (Table 1). Taken together, these two experiments lead us to conclude that LFG is acting in a Fas independent manner in the context of protecting T cells from infection induced apoptosis.

**Table 1:** Apoptosis induced by Jo2.

**%AnnexinV/CD44hi/CD8+**	**IgG**	**Jo2**
WT Day 0 p.i.	33.74±2.16	34.14±2.45
KO Day 0 p.i.	37.54±3.74	38.77±3.56
WT Day 2 p.i.	38.37±1.58	48.35±1.83*
KO Day 2 p.i.	51.82±0.74**	60.17±1.26* ^,^ **
**%AnnexinV/CD44hi/CD4+**	**IgG**	**Jo2**
WT Day 0 p.i.	52.46±2.31	63.7±1.28*
KO Day 0 p.i.	57.26±2.46	67.43±1.33*^,^ **
WT Day 2 p.i.	60.37±1.39	69.27±0.84*
KO Day 2 p.i.	68.37±0.8**	74.45±1.05* ^,^ **

This table represents the percentage of AnnexinV^+^ WT and KO CD44^hi^CD8 and CD44^hi^CD4 cells after culturing the splenocytes of naïve (day 0) or day 2 p.i. chimaeric mice in the presence of either IgG control or the Fas agonist Jo2 for 16 hours. (n = 4 mice per timepoint, ± = SEM).

*p ≤ 0.05 between IgG and Jo2 conditions

**p ≤ 0.05 between WT and KO under the IgG or Jo2 condition

## Discussion

We report here the novel finding that LFG plays a role in protecting T cells from apoptosis during antiviral immune response. The inflammatory environment of the first few days of an infection is especially hostile for antigen experienced T cells. In this context we observed a marked increase in apoptosis of LFG KO CD44^hi^ cells., most of which are pre-existing memory T cells, therefore we can conclude that LFG is involved in memory T cell survival during the attrition process. However, the question is if newly activated T cells in response to the LCMV virus are also affected by the loss of LFG.

Our data showed a diminished immune response of the LFG KO T cells (Fig 2A) with the final outcome of a reduced LCMV specific memory pool (Fig 2B). This can be caused by: 1) Reduced proliferation of the LFG KO T cells, which we can rule out with the *in vivo* BrdU data (Fig 3A) and the *in vitro* CFSE study (S1 Fig).

2) Increased apoptosis of newly activated T cells which is supported by the increased apoptosis found in CD44hi KO T cells in the early phase of the viral infection and the fact that LFG expression is increased when T cells are activated with CD3/CD28.

Therefore, we hypothesize that LFG may be involved in the survival of recently activated T cells in response to LCMV infection. In order to prove our hypothesis we would need to determine if virus specific T cells at days 2 and 3 are dying in the absence of LFG, unfortunately it is too early to detect interferon gamma production in these cells.

In the case that there was a reduced number of newly activated T cells in the LFG KO pool it would be almost indistinguishable at first, but the exponential proliferation during the expansion phase would greatly amplify any small differences, resulting in the 3 fold reduction of the amount of LCMV specific LFG KO CD8 memory T cells (Fig 2B).

These results may implicate a role for LFG in vaccine designs with inflammatory adjuvant, where a 50% reduction in memory T cells can result in a drop below the critical threshold for effective secondary response. It would be interesting to know in future studies whether over expression of LFG can boost memory T cell levels.

The exact mechanism of LFG’s protection from apoptosis has been elusive to us and other labs that are studying the LFG functions [10]. Since it was discovered as an inhibitor of FAS mediated apoptosis, all studies on LFG have been focused on its interactions with players of that pathway. However, we were surprised to find that blocking the FAS pathway did not rescue the early apoptosis in CD44^hi^CD8 T cells in LFG KO mice. Previous studies have shown that FAS pathway does not contribute to the early attrition of memory CD8 T cells in wild type mice [11], but we expected that blocking FAS would inhibit the additional apoptosis of CD8 T cells in LFG-KO cells. Our FAS blocking, as well as Caspase 8 inhibition were effective *in vivo* because they blocked 50% of the early CD44^hi^CD4 apoptosis, agreeing with the consensus in literature that CD4 memory cells are more sensitive to FAS mediated apoptosis, however, the similar level of rescue between WT and KO mice implicated that FAS alone was not responsible for the increased apoptosis in CD4 memory T cells either.

There are several published observations that suggest the possibility of LFG’s involvement in the intrinsic pathway. First, sequence analysis revealed that LFG shares a C terminus motif and structurally resembles the BAX inhibitory protein (BI-1) that resides in the endoplasmic reticulum (ER) and protects cells against ER stress induced apoptosis [12,13]. LFG apart from the plasma membrane is also present in the ER [1] and it has been shown to interact with Bax by co-immunoprecipitation [12].

In addition Bcl2 and Bax are also expressed in the ER providing a location for the interaction of these proteins [14,15]. Therefore, it is possible that LFG’s interaction with Bax is inhibiting its function and releasing the inhibitory effect on Bcl2 in order to protect cells from apoptosis. We think it would be important for future work to further investigate the role of LFG in the intrinsic cell death pathways, because the list of proteins that can mediate crosstalk between the intrinsic and extrinsic pathways is quite short.

## Supporting Information

S1 Fig Similar proliferative response of WT and KO T cells to CD3 stimulation. Splenocytes from WT or KO mice were CFSE labeled and stimulated with plate-bound CD3 and soluble CD28 for 1, 2 or 3 days. Unstimulated CD8 (Column 1) and CD4 (Column 3) did not proliferate, while stimulated CD8 (Column 2) and CD4 (Column 4) T cells proliferated abundantly. However, there are no differences in the pattern of proliferation between WT and KO cells.
(TIF)
