# Supplementary figures and images for: Antiapoptotic Role for Lifeguard in T Cell Mediated Immune Response
Source: PLoS One. 2015 Nov 13;10(11):e0142161. doi: 10.1371/journal.pone.0142161 (PMC4643990; doi:10.1371/journal.pone.0142161)

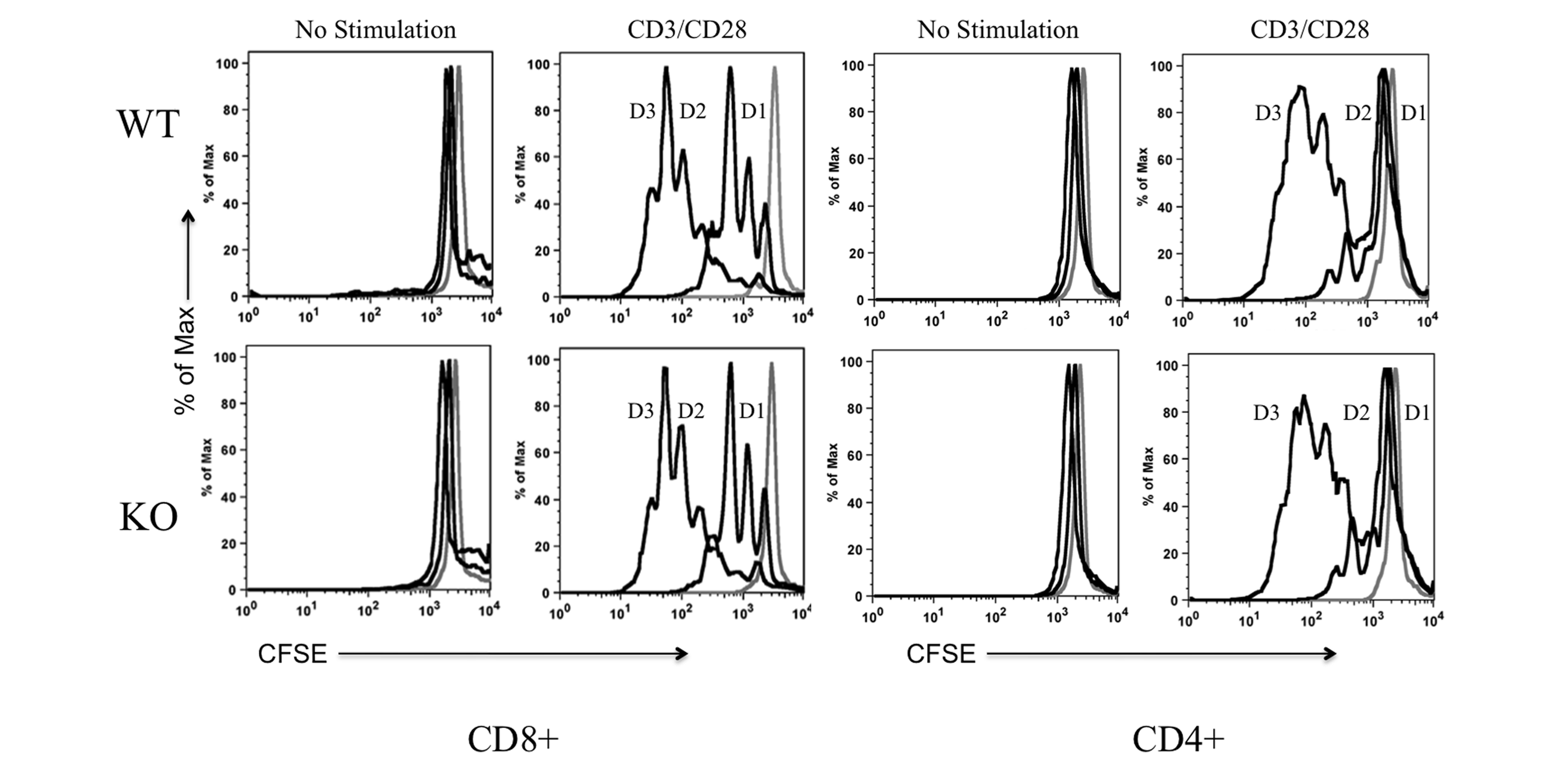

Supplement: S1 Fig — Splenocytes from WT or KO mice were CFSE labeled and stimulated with plate-bound CD3 and soluble CD28 for 1, 2 or 3 days. Unstimulated CD8 (Column 1) and CD4 (Column 3) did not proliferate, while stimulated CD8 (Column 2) and CD4 (Column 4) T cells proliferated abundantly. However, there are no differences in the pattern of proliferation between WT and KO cells. (TIF) [file pone.0142161.s001.tif]
